# Pleomorphic adenomas and mucoepidermoid carcinomas of the breast are underpinned by fusion genes

**DOI:** 10.1038/s41523-020-0164-0

**Published:** 2020-06-05

**Authors:** Fresia Pareja, Arnaud Da Cruz Paula, Rodrigo Gularte-Mérida, Mahsa Vahdatinia, Anqi Li, Felipe C. Geyer, Edaise M. da Silva, Gouri Nanjangud, Hannah Y. Wen, Zsuzsanna Varga, Edi Brogi, Emad A. Rakha, Britta Weigelt, Jorge S. Reis-Filho

**Affiliations:** 1 Department of Pathology, Memorial Sloan Kettering Cancer Center, New York, NY USA; 2 Department of Surgery, Memorial Sloan Kettering Cancer Center, New York, NY USA; 3 Molecular Cytogenetics Core Facility, Memorial Sloan Kettering Cancer Center, New York, NY USA; 4 Institute of Pathology and Molecular Pathology, University Hospital Zurich, Zurich, Switzerland; 5 Department of Pathology, Nottingham University, Nottingham, UK

**Keywords:** Breast cancer, Breast cancer

## Abstract

Primary pleomorphic adenomas (PAs) and mucoepidermoid carcinomas (MECs) of the breast are vanishingly rare. Here we sought to determine whether breast PAs and MECs would be underpinned by the fusion genes reported to occur in their salivary gland counterparts. Our study included three breast PAs and one breast MEC, which were subjected to RNA sequencing (PAs, *n* = 2; MEC, *n* = 1) or to Archer FusionPlex sequencing (PA, *n* = 1). Our analyses revealed the presence of the *HMGA2*-*WIF1* fusion gene in breast PA3, the *CTNNB1*-*PLAG1* fusion gene in breast PA2, and the *CRTC1*-*MAML2* fusion gene in the breast MEC analyzed (1/1). No oncogenic fusion genes were detected in breast PA1, and no additional oncogenic fusion genes were detected in the cases studied. The presence of the fusion genes identified was validated by fluorescence in situ hybridization (*n* = 1), reverse transcription-PCR (*n* = 1), or by both methods (*n* = 1). Taken together, our findings indicate that PAs and MECs arising in the breast resemble their salivary gland counterparts not only phenotypically but also at the genetic level. Furthermore, our data suggest that the molecular analysis of breast PAs and MECs might constitute a useful tool to aid in their differential diagnosis.

## Introduction

Pleomorphic adenomas (PAs) and mucoepidermoid carcinomas (MECs) of the breast are rare tumors that histologically resemble their salivary gland counterparts^1^. PAs are characterized by an admixture of epithelial and myoepithelial cells immersed in myxochondroid stroma^1,2^. Cartilaginous and osseous metaplasia may be present^1,2^. Breast PAs usually have a benign course, although local recurrences have been described^1^. PAs arising in the salivary glands, skin, and soft tissue harbor recurrent gene rearrangements involving *PLAG1* or *HMGA2* (refs. ^3,4^). MECs, although common in the salivary glands, are vanishingly rare in the breast^1,5^. MECs are characterized by the admixture of different cell types, including mucinous, intermediate, and squamous cells^1^. MECs arising in the salivary gland, lung, and cervix have been shown to harbor the t(11,19)(q14–21; p12–13) translocation that results in the *CRTC1*-*MAML2* fusion gene^6–8^, or less frequently the *CRTC3-MAML2* fusion gene^9^.

A subset of salivary gland-like tumors arising in the breast have been shown to be underpinned by the same genetic alterations as their salivary glands counterparts^1,10^. For instance, adenoid cystic carcinomas arising either in the breast or in the salivary glands harbor *MYB* or *MYBL1* rearrangements, regardless of their anatomic origin^10–12^, and secretory carcinomas of the breast and mammary analog secretory carcinomas of the salivary glands are characterized by the *ETV6*-*NTRK3* fusion gene^10,13,14^. The genetic underpinning of other breast salivary gland-like tumors, such as acinic cell carcinomas, however, is different than that of their salivary gland counterparts. Salivary gland acinic cell carcinomas are characterized by the t(4;9)(q13;q31) translocation, which results in a rearrangement involving the nuclear transcription factor *NR4A3* (ref. ^15^), whereas breast acinic cell carcinomas seem not to harbor a pathognomonic genetic alteration and consistently display complex genomes and *TP53* mutations^16,17^. Here we sought to determine whether breast PAs and MECs arising in the breast would be underpinned by fusion genes, in particular those reported in their salivary gland counterparts.

## Results

### Cases

**Fig. 1:**
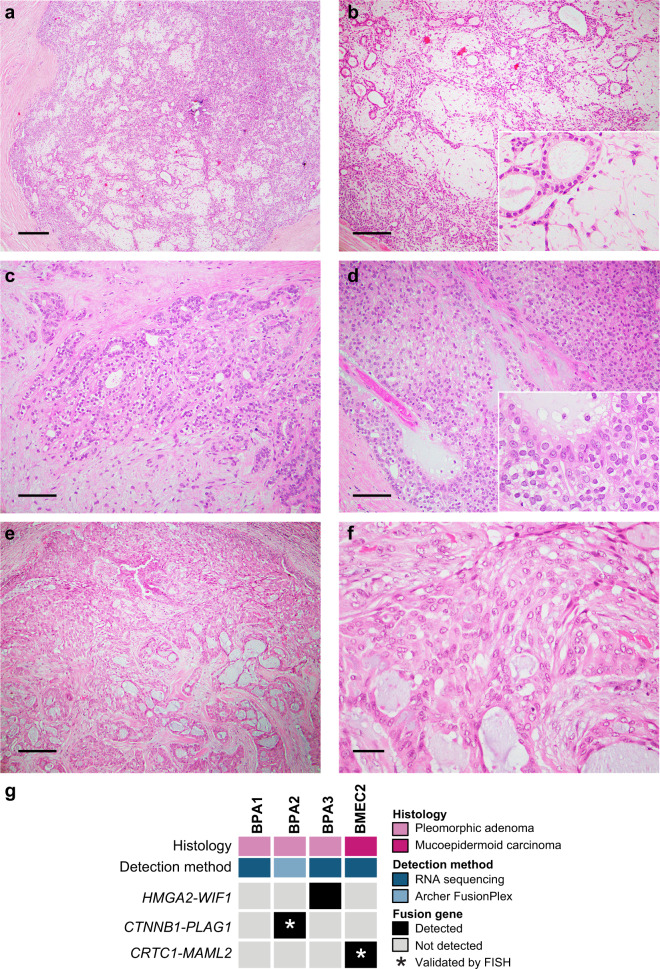
Histologic features of and fusion genes in breast pleomorphic adenomas and a mucoepidermoid carcinoma. **a**–**d** Representative photomicrographs of hematoxylin and eosin (H&E)-stained sections of breast pleomorphic adenomas (PAs) included in this study. Breast PAs were well-circumscribed lesions (**a**), composed of epithelial and myoepithelial cells in a myxochondroid stroma (**b**, **c**). Focal areas with solid growth were also identified (**d**). **e**, **f** Representative photomicrographs of H&E-stained sections of the breast mucoepidermoid carcinoma (MEC) displaying squamous, mucinous, and intermediate cells (**e**), and absence of atypia or necrosis (**f**). **g** Heatmap depicting fusion genes identified in breast pleomorphic adenomas (BPAs; *n*=3) and a breast mucoepidermoid carcinoma (BMEC2; *n*=1). Cases are shown in columns and fusion genes in rows. Tumor type and detection method are shown (top). Scale bars, 200μm (**a**), 100μm (**b**, **e**), 50μm (**c**, **d**), and 20μm (**f**). FISH fluorescence in situ hybridization.

Three PAs and one MEC arising in the breast were included in this study. The breast PAs (*n* = 3) included in our series were well-circumscribed lesions (Fig. 1a), composed of epithelial cells and myoepithelial cells arranged in cords, clusters, and forming glands immersed in a myxochondroid stroma (Fig. 1b, c). Myoepithelial cells floating in the stroma as single stellate cells (Fig. 1b) and focal areas of solid growth were frequently observed (Fig. 1d). No cellular atypia or increased mitotic index were detected (Fig. 1d) The breast MEC studied here corresponded to a low-grade MEC with well-circumscribed borders (Fig. 1e), composed of mucinous, intermediate, and squamous cells arranged in cords, sheets, and clusters (Fig. 1e). No cytologic atypia or necrosis were observed (Fig. 1e, f).

To determine whether the breast PAs (*n* = 3) and the MEC (*n* = 1) included in our series would harbor fusion genes, akin to their counterparts arising in the salivary glands, we subjected two breast PAs (BPA1 and BPA3) and one MEC (BMEC2) to RNA sequencing (Fig. 1g, Supplementary Table 1). RNA-sequencing data were analyzed using a validated bioinformatics pipeline for *de novo* discovery of fusion genes as previously described^18,19^. RNA derived from one breast PA (BPA2) with insufficient material for RNA sequencing was subjected to Archer FusionPlex analysis, a targeted RNA-based assay, for fusion gene detection (Fig. 1g, Supplementary Table 1). Our analyses revealed the presence of fusion genes in two of the three breast PAs and in the breast MEC analyzed (Fig. 1g, Supplementary Table 1).

### *HMGA2* and *PLAG1* rearrangements in breast pleomorphic adenomas

**Fig. 2:**
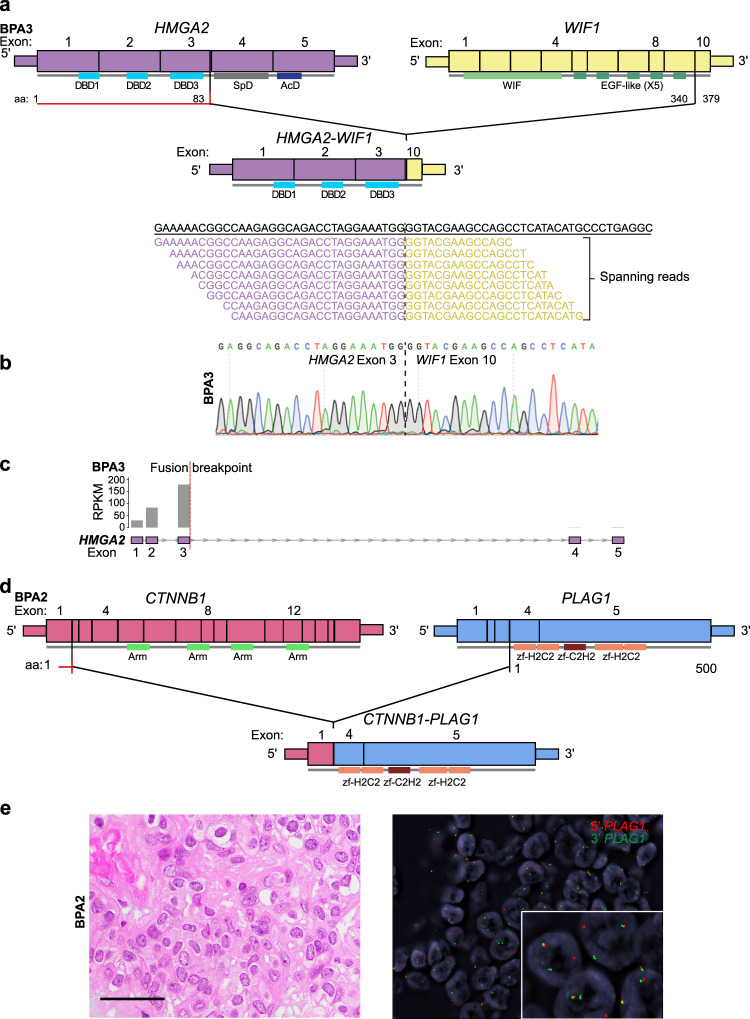
Fusion genes identified in breast pleomorphic adenomas. **a** Schematic representation of the *HMGA2*-*WIF1* fusion transcript detected in the pleomorphic adenoma BPA3 by RNA sequencing, depicting the exons and protein domains involved (top). Vertical lines show the breakpoints of *HMGA2* and *WIF1*. The spanning reads found to cross the genomic breakpoint of the *HMGA2*-*WIF1* chimeric transcript are shown aligned to the predicted junction sequence (bottom). **b** Representative Sanger sequencing electropherogram of cDNA validating the *HMGA2*-*WIF1* fusion junction in BPA3. **c** Schematic representation showing the reads per kilobase per million (RPKM) mapped read counts of each *HMGA2* exon in the pleomorphic adenoma BPA3. The *HMGA2* fusion breakpoint is represented as a red dashed line. **d** Schematic representation of the *CTNNB1*-*PLAG1* fusion transcript identified in the pleomorphic adenoma BPA2 by Archer FusionPlex, depicting the exons and protein domains involved. Vertical lines show the breakpoints of *CTNNB1* and *PLAG1*. **e** Representative photomicrographs of a hematoxylin and eosin-stained section (left) and fluorescence in situ hybridization using *PLAG1* dual-color break-apart probes (red, 5′ *PLAG1*, green, 3′ *PLAG1;* right) of BPA2. Scale bar, 20 μm (**e**). AcD acidic domain, DBD DNA-binding domain, SpD spacer domain, Arm Armadillo repeat, zf-H2C2 Zinc finger double domain, zf-C2H2 Zinc finger C2H2 type.

Our analyses revealed the presence of an *HMGA2-WIF1* fusion gene in BPA3 by RNA sequencing (Figs. 1g and 2a, Supplementary Table 1). The *HMGA2*-*WIF1* fusion gene identified has been previously reported in PAs of the salivary gland^20,21^. *HMGA2* encodes for a transcriptional regulator of the TGF-β signaling pathway^22^, and *WIF1* for a tumor suppressor modulating signaling via the Wnt pathway^20^. The *HMGA2*-*WIF1* fusion gene detected in BPA3 resulted in a chimeric transcript composed of exons 1–3 of *HMGA2* and exon 10 of *WIF1*, and was predicted to be translated in the initial segment of HMGA2 fused to the C′-terminus of WIF1, with the loss of its active WIF domain^20^ (Fig. 2a). The *HMGA2*-*WIF1* fusion gene identified in BPA3 was validated by reverse transcription (RT)-PCR, followed by Sanger sequencing (Fig. 2b). As predicted, the *HMGA2*-*WIF1* fusion resulted in retained expression of *HMGA2* exons 1–3 and decreased expression of exons 4 and 5 (Fig. 2c). We identified the presence of a *CTNNB1*-*PLAG1* fusion gene in BPA2 by Archer FusionPlex targeted RNA sequencing (Figs. 1g and 2d, Supplementary Table 1). The *CTNNB1*-*PLAG1* fusion gene was predicted to result in a chimeric transcript encompassing exon 1 of *CTNNB1* and exons 4 and 5 of *PLAG1* (Fig. 2d) and has been described in PAs of the salivary glands and of the lacrimal glands^23,24^. The *CTNNB1*-*PLAG1* fusion identified in BPA2 was validated by fluorescence in situ hybridization (FISH) using *PLAG1* dual-color break-apart probes (Fig. 2e). No additional likely pathogenic fusion genes were identified in the breast PAs analyzed (Supplementary Tables 1 and 2).

Given that *HMGA2* and *PLAG1* rearrangements are the hallmark genetic alterations of PAs, regardless of their anatomic location^3,4^, we subjected the breast PA included in this study in which no fusion genes were identified by RNA sequencing (BPA1) to FISH analysis using *HMGA2* and *PLAG1* dual-color break-apart probes, which confirmed the absence of *HMGA2* or *PLAG1* rearrangements (Supplementary Table 1). Notably, BPA1 did not differ histologically from the PAs found to harbor fusion genes.

Breast adenomyoepitheliomas and PAs may show histologic and genetic overlap^25^. Indeed, we have reported on a *bona fide* breast adenomyoepithelioma displaying focal myxoid matrix and harboring an *HMGA2*-*WIF1* fusion gene^26^. Hence, we sought to determine whether the breast PAs of this series subjected to RNA sequencing would harbor hotspot mutations^27^ affecting *HRAS*, *PIK3CA*, and *AKT1* as in breast adenomyoepitheliomas^28^. Upon manual inspection of the *HRAS* Q61 hotspot locus, *PIK3CA* H1047, E545, E542, G118, G1050, and G106 hotspot loci and *AKT1* E17 hotspot locus in the RNA sequencing BAM files of BPA1 and BPA3 using the Integrative Genomics Viewer (IGV)^29^, no hotspot mutations affecting these genes were identified.

### *CRTC1-MAML2* fusion gene in a breast mucoepidermoid carcinoma

**Fig. 3:**
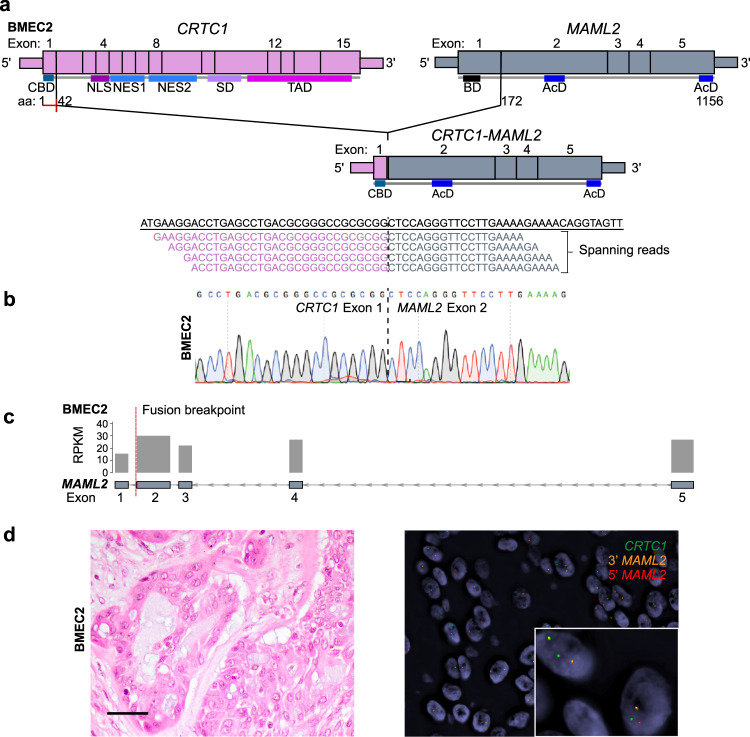
*CRTC1*-*MAML2* fusion gene identified in a breast mucoepidermoid carcinoma. **a** Schematic representation of the *CRTC1*-*MAML2* fusion transcript detected in the breast mucoepidermoid carcinoma BMEC2 by RNA sequencing, depicting the exons and protein domains involved (top). Vertical lines show the breakpoints of *CRTC1* and *MAML2*. The spanning reads found to cross the genomic breakpoint of the *CRTC1*-*MAML2* chimeric transcript are shown aligned to the predicted junction sequence (bottom). **b** Representative Sanger sequencing electropherogram of cDNA validating the *CRTC1*-*MAML2* fusion junction in BMEC2. **c** Schematic representation showing the reads per kilobase per million (RPKM) mapped read counts of each *MAML2* exon. The *MAML2* fusion breakpoint is represented as a red dashed line. **d** Representative photomicrographs of a hematoxylin and eosin-stained section (left) and fluorescence in situ hybridization using three-color break-apart probes (green, *CRTC1*; 5′ *MAML2*, red; 3′ *MAML2*, orange; right) of BMEC2. Scale bar, 20 μm (**d**). AcD acidic domain, BD basic domain, CBD CREB-binding domain, NES nuclear export signal, NLS nuclear localization signal, SD splicing domain, TAD transactivation domain.

RNA sequencing analysis of BMEC2, a MEC arising in the breast, revealed the presence of a *CRTC1*-*MAML2* fusion gene (Figs. 1g and 3a), the hallmark genetic alteration of MECs arising in other anatomic origins^6–8^. These findings are in agreement with the study by Bean et al.^30^, who reported on two breast MECs harboring the *CRTC1*-*MAML2* fusion gene. The fusion gene identified in BMEC2 was predicted to result in a chimeric transcript encompassing exon 1 of *CRTC1* and exons 2–5 of *MAML2* (Fig. 3a, b), akin to MECs in other anatomic locations and to the two breast MECs previously reported^6–8^. As predicted, the *CRTC1-MAML2* fusion resulted in increased expression of exons 2–5 and decreased expression of exon 1 of *MAML2* (Fig. 3c), and its presence was confirmed by RT-PCR and FISH using a three-color break-apart probe (Fig. 3b, d).

## Discussion

Given that various salivary gland-like tumors arising in the breast have been shown to harbor the same genetic alterations as their salivary gland counterparts^10^, we posited that breast PAs and MECs might be underpinned by the oncogenic fusion genes described in PAs and MECs arising in the salivary glands. Our analyses resulted in the identification of an *HMGA2*-*WIF1* and a *CTNNB1*-*PLAG1* fusion gene in breast PAs, and of a *CRTC1*-*MAML2* fusion gene in a breast MEC. The *HMGA2* and *PLAG1* rearrangements, and the *CRTC1*-*MAML2* fusion genes identified in the breast PAs and breast MECs of this study, have been reported in PAs and MECs arising in the salivary gland and in other anatomic locations^6–8,20,21,23,24^. Moreover, our findings on breast MECs are in agreement with the study by Bean et al.^30^, who reported on the presence of the *CRTC1*-*MAML2* fusion gene in two breast MECs.

*HMGA2* is an oncogene with diverse oncogenic mechanisms, as it has been shown to induce the activity of E2F1 and AP1, result in inactivation of p53-dependent apoptosis, and in the activation of the TGF-β signaling pathway^22,31^. WIF1 is a tumor suppressor that encodes for a secreted antagonist that binds Wnt proteins hampering ligand–receptor interactions^32,33^. The *HMGA2*-*WIF1* fusion gene identified in BPA3 results in the loss of the 3′ UTR regulatory sites of *HMGA2*, resulting in its overexpression and in increased signaling via TGF-β^22,34^. It is also predicted to result in the loss of the WIF domain of WIF1, required for its tumor suppressor activities, with ensuing Wnt signaling activation^32,33^. We have recently reported the presence of an *HMGA2*-*WIF1* fusion gene in an estrogen receptor-positive breast adenomyoepithelioma (AME) lacking *HRAS* mutations^26^. Although the *HMGA2* and *WIF1* genomic breakpoints in the *HMGA2*-*WIF1* fusion gene identified in the *HRAS*-wild type AME were different than those identified in BPA3, they resulted in the loss of the 3′UTR of *HMGA2* and of the WIF domain of *WIF1* (ref. ^26^), akin to the fusion gene present in BPA3 reported here. Notably, the previously reported AME harboring the *HMGA2*-*WIF1* fusion gene^26^, despite having the cardinal diagnostic features of AMEs, displayed focal myxochondroid stroma, akin to that found in PAs^26^. We identified a *CTNNB1*-*PLAG1* fusion gene in BPA2, described in PAs of the salivary gland. This fusion gene results in promoter swapping between *PLAG1* and *CTNNB1*, leading to increased *PLAG1* expression and reduction of *CTNNB1* expression^35^. Taken together, these findings suggest that the spectrum of histologic features of breast PAs and AMEs overlaps, despite the differences in their repertoire of somatic genetic alterations (i.e. *HMGA2* and *PLAG1* fusion genes in the majority of PAs, in contrast with mutations affecting *HRAS, PIK3CA, AKT1*, and *PIK3R1* in breast AMEs)^3,4,28^.

MECs of the salivary glands and two breast MECs previously reported are characterized by the t(11:19) (q21;p13) translocation, which results in the *CRTC1*-*MAML2* fusion gene^6,30^. *CRTC1* encodes for the CREB-regulated transcriptional coactivator 1 (ref. ^36^) and *MAML2* for the NOTCH/RBPJ mastermind-like 2. MAML2 forms DNA-binding complexes with Rbp-j and NotchIC and augments Notch signaling^37^. The *CRTC1*-*MAML2* fusion gene identified in BMEC2 has been shown to result in activation of the Notch signaling pathway, be tumorigenic in vitro, and silencing of its fusion product reportedly inhibits tumor growth^38^.

Given their rarity, the differential diagnosis of PAs and MECs arising in the breast from other breast tumors, such as metaplastic carcinomas and squamous cell carcinomas, respectively, might be challenging^1^. The absence of peripheral myoepithelial cell layer between the epithelium and stroma in PAs together with their infiltrative appearances, at least focally, makes their distinction from metaplastic carcinoma of the matrix producing type challenging, particularly in core biopsies. Such distinction may have significant management implications. Similarly, the distinction between MECs and the more aggressive metaplastic squamous cell carcinoma or the benign papilloma with squamous metaplasia can be difficult and an additional genomic testing may help in their accurate diagnosis. Reanalysis of the RNA sequencing results by Piscuoglio et al.^39^ and Weigelt et al.^40^ revealed that none of the metaplastic breast cancers analyzed in those studies harbored any of the fusion genes identified in breast PAs and MECs (data not shown). Our findings suggest that the assessment of rearrangements involving *PLAG1* or *HMGA2* in breast PAs, and of *MAML2* rearrangements in breast MECs, might be used as ancillary tools to aid in the diagnosis of these rare breast tumors.

Our study has important limitations, including the small sample size due to the rarity of PAs and MECs arising in the breast. In addition, we could only perform transcriptomic analyses of the samples included here due to limited available material. Finally, no fusion genes were identified in one of the breast PAs analyzed, and its driver has yet to be identified. Despite these limitations, our findings support the notion that PAs and MECs arising in the breast harbor genetic alterations akin to those described in their salivary gland counterparts, and constitute additional examples of genotypic–phenotypic correlations in breast tumors. These findings may also constitute the basis for the development of ancillary methods for the diagnosis of these tumors in the breast.

## Methods

### Ethics

This study was approved by the Memorial Sloan Kettering Cancer Center Institutional Review Board (IRB) and local research ethics committees of the authors’ institutions. Patient consent was obtained if required by the approved IRB protocols. Formalin-fixed paraffin-embedded (FFPE) tissue blocks of three breast PAs and one breast MEC were retrieved from the pathology archives of the authors’ institutions. All samples were anonymized before analysis.

### Cases and RNA extraction

All tumors included in this study were centrally reviewed by four pathologists (F.P., M.V., F.C.G., J.S.R.-F.) for diagnosis confirmation, following the diagnostic criteria put forward by the World Health Organization (WHO)^41^. The three breast PAs and one breast MEC were microdissected from eight-micron-thick histologic sections under a stereomicroscope (Olympus SZ61) to ensure a tumor cell content >80%, and RNA was extracted using the RNeasy FFPE Kit.

### RNA sequencing and identification of fusion genes

FFPE-derived RNA from two breast PA and one breast MEC were subjected to paired-end RNA sequencing following the standard protocols used at the Integrated Genomics Operation at MSKCC^42^, as previously described^19,26,43^. INTEGRATE^44^, deFuse^45^, and FusionCatcher^46^ were used to identify read pairs supporting chimeric transcripts, followed by exclusion of candidate fusion transcripts and read-through candidates identified in a set of 297 normal breast tissues from the TCGA^47^. The Bayesian probability to constitute drivers of the remaining candidate fusion genes supported by at least two spanning reads was assessed using OncoFuse (v1.0.9b2)^48^. Reads per kilobase million (RPKM) values were calculated from raw counts after normalization for sequencing depth and following adjustment for length of genes. Data are available as described on the Data availability statement^49,50^.

### ARCHER FusionPlex

One breast PA with insufficient material for RNA sequencing was subjected to Archer FusionPlex assay, an RNA-based panel that uses Archer Anchored Multiplex PCT technology and next-generation sequencing for the identification of fusion genes^51^ at the Integrated Genomics Operation at MSKCC, as previously described^52^. Archer FusionPlex data were analyzed using Archer Software (v4.0.10).

### RT-PCR for fusion gene validation

RNA was reverse-transcribed using SuperScript VILO Master Mix (ThermoFisher Scientific), following the manufacturers’ instructions. PCR amplification of 10 ng cDNA was conducted using fusion gene-specific primers sets designed based on the identified breakpoints (Supplementary Table 3). PCR fragments were purified with ExoSAP-IT (ThermoFisher Scientific) and subjected to Sanger Sequencing. Sequence electropherograms of the forward and reversed strands were manually inspected.

### Fluorescence in situ hybridization

Cases in this study with available material were subjected to FISH analysis for rearrangements involving *HMGA2* and *PLAG1* or for the presence of the *CRTC1*-*MAML2* fusion gene following validated protocols at the Molecular Cytogenetics Core of MSKCC, as previously described^19^. *HMGA2* and *PLAG1* rearrangements were assessed using dual-color break-apart probes consisting of bacterial artificial chromosome (BAC) clones mapping to 5′ *HMGA2* (RP11-230G5, RP11-662G15, red) and 3′ *HMGA2* (RP11-937C6, RP11-167E10, green) and to 5′ *PLAG1* (RP11-92A9, RP11-111I18, red) and 3′ *PLAG1* (RP11-144E19, RP11-246A9, green), respectively. The presence of *CRTC1*-*MAML2* fusion gene was assessed using a three-color break-apart probe consisting of BAC clones mapping to *CRTC1* (RP11-282P1, RP11-908B10, green), 5′*MAML2* (RP11-385G6, RP11-8N17, orange), and 3′*MAML2* (RP11-277H22, RP11-111I14, orange). FISH analysis was conducted by observers blinded to the results of the RNA sequencing and ARCHER FusionPlex analyses. Cases were considered positive if the rearrangement was identified in >15% tumor cells.

### Reporting summary

Further information on research design is available in the Nature Research Reporting Summary linked to this article.

## Supplementary information

Supplementary materials

Reporting Summary

## Data Availability

The data generated and analyzed during this study are described in the following data record: 10.6084/m9.figshare.12199781 (ref. ^49^). The RNA sequencing data have been deposited in the NCBI Sequence Read Archive under the project accession number SRP257886 (https://identifiers.org/ncbi/insdc.sra:SRP257886)^50^. The histologic images, fluorescence in situ hybridization, and Sanger sequencing data will be provided upon reasonable request to Dr. Fresia Pareja. The Archer FusionPlex data will be provided upon reasonable request to Dr. Arnaud Da Cruz Paula.

## References

[1] Foschini MP (2017). The morphological spectrum of salivary gland type tumours of the breast. Pathology.

[2] Pia-Foschini M, Reis-Filho JS, Eusebi V, Lakhani SR (2003). Salivary gland-like tumours of the breast: surgical and molecular pathology. J. Clin. Pathol..

[3] Stenman G (2013). Fusion oncogenes in salivary gland tumors: molecular and clinical consequences. Head. Neck Pathol..

[4] Bahrami A, Dalton JD, Krane JF, Fletcher CD (2012). A subset of cutaneous and soft tissue mixed tumors are genetically linked to their salivary gland counterpart. Genes Chromosomes Cancer.

[5] Basbug M (2011). Mucoepidermoid carcinoma in a breast affected by burn scars: comprehensive literature review and case report. Breast Care (Basel).

[6] Tonon G (2003). t(11;19)(q21;p13) translocation in mucoepidermoid carcinoma creates a novel fusion product that disrupts a Notch signaling pathway. Nat. Genet..

[7] Behboudi A (2006). Molecular classification of mucoepidermoid carcinomas-prognostic significance of the MECT1-MAML2 fusion oncogene. Genes Chromosomes Cancer.

[8] Lennerz JK, Perry A, Mills JC, Huettner PC, Pfeifer JD (2009). Mucoepidermoid carcinoma of the cervix: another tumor with the t(11;19)-associated CRTC1-MAML2 gene fusion. Am. J. Surg. Pathol..

[9] Fehr A (2008). A new type of MAML2 fusion in mucoepidermoid carcinoma. Genes Chromosomes Cancer.

[10] Pareja F (2016). Triple-negative breast cancer: the importance of molecular and histologic subtyping, and recognition of low-grade variants. NPJ Breast Cancer.

[11] Persson M (2009). Recurrent fusion of MYB and NFIB transcription factor genes in carcinomas of the breast and head and neck. Proc. Natl. Acad. Sci. USA.

[12] Kim J (2018). MYBL1 rearrangements and MYB amplification in breast adenoid cystic carcinomas lacking the MYB-NFIB fusion gene. J. Pathol..

[13] Tognon C (2002). Expression of the ETV6-NTRK3 gene fusion as a primary event in human secretory breast carcinoma. Cancer Cell.

[14] Skalova A (2010). Mammary analogue secretory carcinoma of salivary glands, containing the ETV6-NTRK3 fusion gene: a hitherto undescribed salivary gland tumor entity. Am. J. Surg. Pathol..

[15] Haller F (2019). Enhancer hijacking activates oncogenic transcription factor NR4A3 in acinic cell carcinomas of the salivary glands. Nat. Commun..

[16] Beca F (2019). Whole-exome sequencing and RNA sequencing analyses of acinic cell carcinomas of the breast. Histopathology.

[17] Guerini-Rocco E (2015). The repertoire of somatic genetic alterations of acinic cell carcinomas of the breast: an exploratory, hypothesis-generating study. J. Pathol..

[18] Pareja F (2019). The Genomic landscape of mucinous breast cancer. J. Natl Cancer Inst..

[19] Kim SH (2020). Identification of recurrent FHL2-GLI2 oncogenic fusion in sclerosing stromal tumors of the ovary. Nat. Commun..

[20] Queimado L, Lopes CS, Reis AM (2007). WIF1, an inhibitor of the Wnt pathway, is rearranged in salivary gland tumors. Genes Chromosomes Cancer.

[21] Stenman G, Andersson MK, Andren Y (2010). New tricks from an old oncogene: gene fusion and copy number alterations of MYB in human cancer. Cell Cycle.

[22] Fusco A, Fedele M (2007). Roles of HMGA proteins in cancer. Nat. Rev. Cancer.

[23] Kas K (1997). Promoter swapping between the genes for a novel zinc finger protein and beta-catenin in pleiomorphic adenomas with t(3;8)(p21;q12) translocations. Nat. Genet..

[24] Asahina M (2019). Clinicopathological effect of PLAG1 fusion genes in pleomorphic adenoma and carcinoma ex pleomorphic adenoma with special emphasis on histological features. Histopathology.

[25] McLaren BK, Smith J, Schuyler PA, Dupont WD, Page DL (2005). Adenomyoepithelioma: clinical, histologic, and immunohistologic evaluation of a series of related lesions. Am. J. Surg. Pathol..

[26] Pareja F (2019). Assessment of HMGA2 and PLAG1 rearrangements in breast adenomyoepitheliomas. NPJ Breast Cancer.

[27] Chang MT (2016). Identifying recurrent mutations in cancer reveals widespread lineage diversity and mutational specificity. Nat. Biotechnol..

[28] Geyer FC (2018). Recurrent hotspot mutations in HRAS Q61 and PI3K-AKT pathway genes as drivers of breast adenomyoepitheliomas. Nat. Commun..

[29] Thorvaldsdottir H, Robinson JT, Mesirov JP (2013). Integrative Genomics Viewer (IGV): high-performance genomics data visualization and exploration. Brief. Bioinformatics.

[30] Bean GR (2019). CRTC1-MAML2 fusion in mucoepidermoid carcinoma of the breast. Histopathology.

[31] Morishita A (2013). HMGA2 is a driver of tumor metastasis. Cancer Res..

[32] Malinauskas T, Aricescu AR, Lu W, Siebold C, Jones EY (2011). Modular mechanism of Wnt signaling inhibition by Wnt inhibitory factor 1. Nat. Struct. Mol. Biol..

[33] Hsieh JC (1999). A new secreted protein that binds to Wnt proteins and inhibits their activities. Nature.

[34] Mayr C, Hemann MT, Bartel DP (2007). Disrupting the pairing between let-7 and Hmga2 enhances oncogenic transformation. Science.

[35] Astrom AK (1999). Conserved mechanism of PLAG1 activation in salivary gland tumors with and without chromosome 8q12 abnormalities: identification of SII as a new fusion partner gene. Cancer Res..

[36] Conkright MD (2003). TORCs: transducers of regulated CREB activity. Mol. Cell.

[37] Kitagawa M (2016). Notch signalling in the nucleus: roles of Mastermind-like (MAML) transcriptional coactivators. J. Biochem..

[38] Komiya T (2006). Sustained expression of Mect1-Maml2 is essential for tumor cell growth in salivary gland cancers carrying the t(11;19) translocation. Oncogene.

[39] Piscuoglio S (2017). Genomic and transcriptomic heterogeneity in metaplastic carcinomas of the breast. NPJ Breast Cancer.

[40] Weigelt B (2015). Metaplastic breast carcinomas display genomic and transcriptomic heterogeneity [corrected]. Mod. Pathol..

[41] WHO Classification of Tumors Editorial Board. (2019). Breast Tumours. WHO Classification of Tumors..

[42] Weinreb I (2014). Hotspot activating PRKD1 somatic mutations in polymorphous low-grade adenocarcinomas of the salivary glands. Nat. Genet..

[43] Pareja F (2018). Loss-of-function mutations in ATP6AP1 and ATP6AP2 in granular cell tumors. Nat. Commun..

[44] Zhang J (2016). INTEGRATE: gene fusion discovery using whole genome and transcriptome data. Genome Res..

[45] McPherson A (2011). deFuse: an algorithm for gene fusion discovery in tumor RNA-Seq data. PLoS Comput. Biol..

[46] Edgren H (2011). Identification of fusion genes in breast cancer by paired-end RNA-sequencing. Genome Biol..

[47] Cancer Genome Atlas N. (2012). Comprehensive molecular portraits of human breast tumours. Nature.

[48] Shugay M, Ortiz de Mendibil I, Vizmanos JL, Novo FJ (2013). Oncofuse: a computational framework for the prediction of the oncogenic potential of gene fusions. Bioinformatics.

[49] Pareja F (2020). Pleomorphic adenomas and mucoepidermoid carcinomas of the breast are underpinned by fusion genes. figshare..

[50] NCBI Sequence Read Archive. https://identifiers.org/ncbi/insdc.sra:SRP257886 (2020).

[51] Zheng Z (2014). Anchored multiplex PCR for targeted next-generation sequencing. Nat. Med..

[52] Arias-Stella JA (2019). Novel PLAG1 gene rearrangement distinguishes a subset of uterine myxoid leiomyosarcoma from other uterine myxoid mesenchymal tumors. Am. J. Surg. Pathol..

