# Supplementary material for: Pleomorphic adenomas and mucoepidermoid carcinomas of the breast are underpinned by fusion genes
Source: NPJ Breast Cancer. 2020 Jun 5;6:20. doi: 10.1038/s41523-020-0164-0 (PMC7275089; doi:10.1038/s41523-020-0164-0)
Supplement: Supplementary file 1 — Supplementary materials [file 41523_2020_164_MOESM1_ESM.pdf]

SUPPLEMENTARY MATERIALS

Pleomorphic adenomas and mucoepidermoid carcinomas of the breast are underpinned by fusion genes

Pareja et al.

Supplementary Tables 1-3

Supplementary Table 1. Fusion genes identified in three breast pleomorphic adenomas and a breast mucoepidermoid carcinoma.

Case ID	Histology	Oncogenic fusion genes identified by RNA-sequencing	Oncogenic fusion gene identified by Archer FusionPlex	***PLAG1/HMGA2*** **rearrangement detected by FISH**	***CRTC1-MAML2*** **fusion gene detected by FISH**
BPA1	Pleomorphic adenoma	None	NT	No	NT
BPA2	Pleomorphic adenoma	NT	*CTNNB-PLAG1*	Yes *(PLAG1)*	NT
BPA3	Pleomorphic adenoma	*HMGA2-WIF1*	NT	NT	NT
BMEC2	Mucoepidermoid carcinoma	*CRTC1-MAML2*	NT	NT	Yes

FISH, fluorescence *in situ* hybridization; NT, not tested.

Supplementary Table 2: Fusion candidate genes identified by RNA-sequencing and Archer FusionPlex in the breast pleomorphic adenomas and mucoepidermoid carcinoma included in this study.

Case ID	Fusion Caller (RNA-seq)/Archer FusionPlex	5' Gene	3' Gene	5' Mapping	3' Mapping	Fusion Type	Crossing Reads	Encompassing Reads	In-Frame	Driver Probability (Oncofuse)
BPA3	STAR-Integrate, FusionCatcher, Defuse	*WIF1*	*HMGA2*	12:66232350+	12:66232349-	Intra-Chromosomal	56	145	No	0.024531268
BMEC2	STAR-Integrate, FusionCatcher, Defuse	*CRTC1*	*MAML2*	19:18794638+	11:95826681-	Inter-Chromosomal	4	7	No	0.128278142
BPA2	Archer FusionPlex	*CTNNB1*	*PLAG1*	3:41241161+	8:57083748-	Inter-Chromosomal	6	25	Yes	0.995056115

Supplementary Table 3. Primer sets for validation of candidate fusion genes by RT-PCR

Fusion gene	Primer set	Primer direction	Primer ID	Primer sequence
HMGA2-WIF1	Set #1	Forward	HW-1-Fwd	AATGACTTCCTTTTTCATTTGC
		Reverse	HW-1-Rv	TTAAGTGAAGGCGTGTGCTG
	Set #2	Forward	HW-2-Fwd	AATGTGTCCCTTGGTGCAGT
		Reverse	HW-2-Rv	TTAAGTGAAGGCGTGTGCTG
CRTC1-MAML2	Set #1	Forward	CM-1-Fwd	GCTGCACAATCAGAAGCAG
		Reverse	CM-1-Rv	TGAGTTGTCCACAAAGCCATT
	Set #2	Forward	CM-2-Fwd	CGGAAATTCAGCGAGAAGAT
		Reverse	CM-2-Rv	TGAGTTGTCCACAAAGCCATT
